# Proprioceptive vibration training accelerates independent walking in stroke patients: A retrospective cohort study with survival analysis

**DOI:** 10.1097/MD.0000000000048908

**Published:** 2026-05-22

**Authors:** Hyunsik Yoon

**Affiliations:** a Department of Physical Therapy, College of Health Sciences, Kyungnam University, Changwon-si, Republic of South Korea.

**Keywords:** proprioceptive vibration training, somatosensory evoked potential, stroke

## Abstract

Stroke often leads to persistent impairments in motor function, balance, and gait. Proprioceptive vibration training (PVT) may facilitate sensorimotor integration and functional recovery in individuals with neurological impairment; however, its association with the timing of independent walking after stroke remains unclear. In this retrospective, non-randomized longitudinal cohort study, 102 patients with stroke were categorized into a PVT group (n = 50) or a conventional physical therapy (CPT) group (n = 52). Both groups received 30-minute sessions, 5 d/wk, for 8 weeks. Outcomes included the Fugl-Meyer Assessment–Lower Extremity, Trunk Impairment Scale, Berg Balance Scale (BBS), Functional Ambulation Category (FAC), and Modified Barthel Index. Kaplan–Meier analysis and adjusted Cox proportional hazards regression were used to evaluate time to achieve BBS ≥ 40 and independent walking, defined as FAC ≥ 4. Post-intervention, the proprioceptive vibration training group exhibited significantly higher Berg Balance Scale scores than the conventional physical therapy group (*P* = .02) and Functional Ambulation Category scores (*P* = .01). Survival analysis revealed that the proprioceptive vibration training group achieved independent walking earlier than the conventional physical therapy group (*P* = .01). Adjusted Cox regression showed that proprioceptive vibration training was significantly associated with earlier achievement of independent walking (*P* = .02). PVT combined with conventional training was associated with improved balance and gait ability and with earlier achievement of independent walking in patients with stroke, suggesting its potential clinical utility for functional walking recovery.

## 1. Introduction

Stroke is a leading cause of long-term disability worldwide, frequently resulting in impairments in motor function, balance, gait, and activities of daily living (ADL). These functional deficits substantially impact the autonomy, social participation, and quality of life of stroke survivors.^[1]^ Among various rehabilitation goals, the restoration of independent ambulation is considered one of the most critical milestones, as it facilitates greater independence and faster reintegration into daily life and the community.^[2]^

Traditional rehabilitation approaches typically focus on restoring motor function using neurodevelopmental techniques and task-specific training. While effective to some degree, these methods may be insufficient to fully address sensorimotor integration and postural control, particularly in individuals with neurological impairments.^[3]^ In response, recent advances in neurorehabilitation have highlighted the potential benefits of proprioceptive stimulation-based interventions, such as proprioceptive vibration training (PVT).^[4]^

Proprioceptive vibration training involves the application of mechanical vibrations through a specialized platform to stimulate proprioceptive receptors, including muscle spindles and Golgi tendon organs. Vibration-based interventions can be categorized into focal vibration (targeting specific muscles or tendons) and whole-body vibration (WBV), which delivers mechanical oscillations through a platform to stimulate the entire sensorimotor system. The present study employed WBV-based proprioceptive stimulation, which differs mechanistically from focal vibration in terms of amplitude, frequency, and systemic neuromuscular response.^[5]^ This stimulation may enhance neuromuscular coordination by influencing both spinal reflexes and supraspinal circuits, ultimately facilitating motor recovery and neuroplasticity.^[6]^ Previous studies have shown that vibration-based interventions can improve balance, gait performance, and postural stability in individuals with neurological deficits. However, most of these studies have focused on short-term outcomes like muscle strength or static balance improvements, with limited investigation into clinically meaningful endpoints such as the achievement and timing of independent walking.^[5,7,8]^

Furthermore, the degree of somatosensory impairment, which commonly accompanies stroke, may significantly influence a patient’s response to proprioceptive interventions. Sensory deficits can disrupt postural stability, gait symmetry, and motor relearning. In this context, somatosensory evoked potentials (SSEPs) serve as a valuable electrophysiological tool to objectively assess the integrity of sensory pathways and may act as a predictive biomarker for functional recovery following stroke.^[9]^ Yet, few studies have explored the relationship between SSEP-based sensory classification and outcomes following PVT.

To address these gaps, the present retrospective, non-randomized longitudinal cohort study was conducted to evaluate the effects of PVT on motor function, balance control, gait speed, and activities of daily living in patients with subacute hemiparetic stroke. In particular, we aimed to determine whether PVT accelerates the time to achieve independent ambulation (FAC ≥ 4) compared to conventional physical therapy (CPT), using Kaplan–Meier survival analysis and Cox proportional hazards regression. Additionally, we examined how baseline sensory status, determined by SSEP, influences the likelihood and timing of walking recovery. By integrating proprioceptive intervention outcomes with sensory function assessment, this study seeks to provide new insights into optimizing personalized rehabilitation strategies in stroke care.

## 2. Materials and methods

### 2.1. Participants

A total of 102 patients diagnosed with subacute hemiparetic stroke were retrospectively identified from medical records at a neurorehabilitation hospital and categorized into either the proprioceptive vibration training (PVT, n = 50) group or the conventional physical therapy (CPT, n = 52) group based on the intervention received during their inpatient rehabilitation. According to a meta-analysis on whole-body vibration training in stroke rehabilitation,^[5]^ the expected standardized mean difference in Berg Balance Scale (BBS) change was approximately 0.55. With a 2-sided α of 0.05 and power of 0.80, the required sample size was estimated as 50 participants per group (total N = 100). All patients were within 12 weeks poststroke onset (PVT: 7.54 ± 2.15 weeks; CPT: 7.79 ± 1.56 weeks) and demonstrated adequate cognitive function to follow instructions, with Mini-Mental State Examination (MMSE) scores ≥ 24 (PVT: 25.62 ± 1.52; CPT: 26.23 ± 1.62).

Inclusion criteria were: first-ever unilateral hemiparetic stroke confirmed by imaging, ability to maintain an independent sitting posture for at least 10 seconds (TIS ≥ 2), and stable medical condition suitable for rehabilitation. Patients were excluded if they had: bilateral, cerebellar, or brainstem lesions, severe visual deficits such as hemianopia, uncontrolled hypertension (SBP > 160 mm Hg or DBP > 100 mm Hg), musculoskeletal or cardiopulmonary conditions that prevented ambulation, or a history of neurosurgical intervention.

Between-group homogeneity of baseline variables, including sex distribution, paretic side, sensory impairment classification, age, height, and weight, was assessed using independent *t* tests. No statistically significant differences were found between groups (all *P* > .05), indicating homogeneity at baseline (Table 1).

**Table 1:** Demographic characteristics of the stroke subjects.

Variable	PVT (n = 50)	CPT (n = 52)	*P*-value
Sex (male/female)	26/ 24	27/ 25	.83
Paretic side (right/left)	31/ 19	29/ 23	.07
Sensory impairment (incomplete/complete)	43/ 7	38/ 14	.10
Onset time (wk)	7.54 ± 2.15	7.79 ± 1.56	.51
Age (yr)	61.28 ± 12.64	65.40 ± 8.40	.06
Height (cm)	165.26 ± 10.67	167.13 ± 8.22	.32
Weight (kg)	64.01 ± 12.64	66.10 ± 12.84	.41
MMSE (score)	25.62 ± 1.52	26.23 ± 1.62	.06

Data are presented as mean ± standard deviation or number of participants.

CPT = conventional physical therapy, MMSE = Mini-mental State Examination, PVT = proprioceptive vibration training.

### 2.2. Materials and procedure

This study was a retrospective, non-randomized longitudinal cohort analysis based on medical records of stroke patients who were hospitalized in the Department of Rehabilitation Medicine at a tertiary general hospital, and for this reason, it was granted an exemption from Institutional Review Board review. All participants received standardized inpatient rehabilitation, including individualized physical and occupational therapy. In addition to standard rehabilitation, the PVT group received an additional 30-minute proprioceptive vibration training program conducted on a vibration platform. In contrast, the control group performed identical strengthening and balance training exercises on a stable support surface.

Pretests were conducted at hospital admission (week 0), and posttests were performed immediately after the 8-week intervention period (week 8). During hospitalization, all patients were assessed weekly from week 1 to week 8. Clinical evaluations were conducted by experienced therapists according to standardized procedures. Functional assessments included lower extremity motor function, balance, gait ability, and activities of daily living. To evaluate somatosensory integrity, somatosensory evoked potential (SSEP) testing was performed. This study was reported in accordance with the STROBE (Strengthening the Reporting of Observational Studies in Epidemiology) statement for observational studies.

#### 2.2.1. Functional ability evaluation

Fugl-Meyer Assessment–Lower Extremity (FMA-LE): A reliable quantitative measure designed to assess motor recovery of the paretic lower limb following stroke. It consists of several items rated on a 3-point scale (0 = cannot perform, 1 = partially performs, 2 = performs fully), with a maximum score of 34 points.^[10]^

Trunk Impairment Scale (TIS): The TIS evaluates static and dynamic sitting balance and trunk coordination. It consists of 3 subscales – static sitting balance, dynamic sitting balance, and trunk coordination – with a maximum score of 23. Higher scores indicate better trunk control. The TIS is widely used in stroke rehabilitation to detect impairments in postural control while sitting.^[11]^

Berg Balance Scale (BBS): A functional balance assessment consisting of 14 items, each rated from 0 to 4 points, with a maximum total of 56. Higher scores reflect better balance. The BBS is highly sensitive to balance changes in stroke patients.^[12]^

Functional Ambulation Category (FAC): The FAC is a 6-level ordinal scale ranging from 0 (nonfunctional ambulation) to 5 (independent ambulation). It classifies walking ability according to the amount of physical assistance or supervision required during ambulation.^[13,14]^ In the present study, independent walking was defined as FAC ≥ 4 because categories 4 and 5 correspond to independent ambulation levels in the original FAC classification. Thus, FAC ≥ 4 was considered a clinically meaningful threshold for functional independent walking.

Modified Barthel Index (MBI): A validated and reliable tool to assess independence in basic ADLs such as feeding, bathing, dressing, toileting, bladder and bowel control, transfers, ambulation, and stair climbing. Total scores range from 0 to 100, with higher scores indicating greater functional independence.^[15]^

#### 2.2.2. Somatosensory evoked potential test

To classify patients according to sensory function prior to rehabilitation, baseline somatosensory evoked potentials (SSEPs) were measured using the Electro Synergy system (Viasys Healthcare, San Diego). Electrical stimulation was applied to the posterior tibial nerve on the paretic side using a bar electrode. The cathode was placed midway between the medial border of the Achilles tendon and the posterior border of the medial malleolus, and the nerve was stimulated at 30 mA. The anode was positioned 2 to 3 cm distal to the cathode.

Electrophysiological recordings were performed according to the international 10 to 20 EEG system. Fine-needle electrodes were inserted at Fz as the reference site and Cz as the active recording site.^[16]^ Stimuli were delivered at a frequency of 2.3 Hz for 250 pulses, and responses were band-pass filtered from 20 to 2000 Hz.^[17]^

Sensory status was classified based on the detectability and latency of the P37 cortical response. The detectability of cortical SSEP responses was used as an indicator of preserved somatosensory pathway integrity. Patients with an identifiable but delayed P37 response, defined as a P37 latency ≥ 41.7 ms, were categorized as having partial sensory impairment, whereas patients with no identifiable P37 waveform were classified as having complete sensory loss.^[18]^ This dichotomous classification was used to distinguish patients with preserved measurable somatosensory pathway responses from those without detectable cortical responses. Based on these criteria, 81 patients were allocated to the partial sensory impairment group, and 21 patients were assigned to the complete sensory loss group.

### 2.3. Intervention

This study was designed to compare the therapeutic effects of conventional physical therapy (CPT) and proprioceptive vibration training (PVT) in patients with stroke. Both groups received 30-minute intervention sessions, 5 d/wk, for 8 weeks. To ensure comparability between groups, the total intervention duration, session frequency, intervention period, and exercise sequence were matched between the CPT and PVT groups. The primary difference between the groups was that the PVT group performed the same exercise program on a side-alternating vibration platform, whereas the CPT group performed the program on a stable support surface.

In the CPT group, participants engaged in a structured physical therapy program under therapist supervision. A therapist certified in neurodevelopmental treatment (NDT) initially adjusted the patient’s sternum, rib cage, and thoracolumbar spine alignment in a standing position to promote proper diaphragmatic breathing. Once the trunk was stabilized, the therapist monitored the patient’s movement patterns and provided corrections as necessary while guiding them through a sequence of exercises.

The exercise program included lower limb strengthening activities, such as repetitive sit-to-stand movements, squats, and quadriceps setting exercises. Weight-bearing activities were incorporated to encourage loading on the paretic limb through forward, backward, and lateral weight shifts. Balance training included static and dynamic standing tasks, such as tandem stance, feet-together stance, and visual-deprivation tasks, including the Romberg stance with eyes closed. Additional exercises targeted single-leg standing balance and stepping coordination, including step-ups and side-steps.^[19]^

The PVT group performed the identical exercise sequence under the same session duration and frequency. However, all exercises were carried out on a vibration platform (Novotec Medical GmbH, Pforzheim, Germany). This side-alternating vibration system was used to provide additional proprioceptive, sensory, and neuromuscular stimulation during the exercise program and to support improvements in functional recovery, strength, and balance.^[20]^ The vibration parameters, including a frequency of 20 to 30 Hz and amplitude of 2.0 to 4.0 mm, were selected based on established methodological recommendations for whole-body vibration therapy in poststroke rehabilitation.^[21]^ These parameters were intended to remain within a therapeutic range for enhancing proprioceptive input, muscle activation, and postural control in individuals with stroke.

### 2.4. Statistical analysis

All statistical analyses were performed using SPSS Statistics for Windows, version 25.0 (IBM Corporation, Armonk). Descriptive statistics were used to summarize baseline characteristics. Data were confirmed to meet the assumptions of normality and homogeneity of variance, as verified by the Shapiro–Wilk and Levene tests, respectively. Independent *t* tests were used to compare post-intervention outcomes between the groups, and paired *t* tests were performed to analyze pre- and post-intervention changes within each group.

Kaplan–Meier survival analysis was conducted to estimate the time to achieve BBS ≥ 40 and independent walking, defined as FAC ≥ 4, and differences between groups were evaluated using the log-rank test. Adjusted Cox proportional hazards regression analysis was applied to identify factors associated with earlier achievement of each endpoint. For the BBS model, age, MMSE, sensory status, and baseline BBS were included as covariates. For the FAC model, age, MMSE, sensory status, and baseline FAC were included as covariates. Group and sensory status were entered as categorical variables, and results were presented as hazard ratios (HRs) with 95% confidence intervals.

## 3. Results

### 3.1. Changes in functional ability (motor function, balance, gait speed, and activities of daily living)

As shown in Table 2, both the PVT group and the CPT group demonstrated improvements in motor function (FMA), sitting balance (TIS), standing balance (BBS), walking ability (FAC), and activities of daily living (MBI) after the intervention.

**Table 2:** Comparison of functional ability between the PVT and CPT groups in stroke patients.

Clinical test		PVT (n = 50)	CPT (n = 52)	*P*-value
FMA-Lower	Pretest	21.12 ± 8.33	19.73 ± 7.68	
	Posttest	26.24 ± 6.49	23.94 ± 7.17	.09
	*t*	−6.87**	−6.70**	
TIS	Pretest	14.30 ± 5.46	13.19 ± 6.03	
	Posttest	19.18 ± 3.81	18.10 ± 4.08	.16
	*t*	−8.12**	−6.91**	
BBS	Pretest	21.86 ± 11.64	21.42 ± 11.92	
	Posttest	40.36 ± 10.73	34.62 ± 13.56	.02*
	*t*	−12.71**	−8.70**	
FAC	Pretest	1.62 ± 1.05	1.63 ± 1.03	
	Posttest	3.28 ± 0.76	2.88 ± 0.83	.01*
	*t*	−11.26**	−10.77**	
MBI	Pretest	46.44 ± 18.55	42.63 ± 17.43	
	Posttest	63.52 ± 18.43	58.55 ± 15.63	.14
	*t*	−11.44**	−9.84**	

Data are presented as mean ± standard deviation. *t* = paired *t* test within group; *P* value = independent *t* test between groups.

BBS = Berg Balance Scale, CPT = conventional physical therapy, FAC = Functional Ambulation Category, FMA = Fugl-Meyer Assessment, MBI = Modified Barthel Index, MMSE = Mini-Mental State Examination, PVT = proprioceptive vibration training, TIS = Trunk Impairment Scale.

* *P* < .05.

** *P* < .01.

The FMA scores increased from 21.12 ± 8.33 to 26.24 ± 6.49 in the PVT group and from 19.73 ± 7.68 to 23.94 ± 7.17 in the CPT group. However, there was no statistically significant difference between groups at posttest (*P* = .09).

The TIS scores improved from 14.30 ± 5.46 to 19.18 ± 3.81 in the PVT group and from 13.19 ± 6.03 to 18.10 ± 4.08 in the CPT group, with no significant difference between groups at posttest (*P* = .169).

The MBI scores improved from 46.44 ± 18.55 to 63.52 ± 18.43 in the PVT group and from 42.63 ± 17.43 to 58.55 ± 15.63 in the CPT group. No significant between-group difference was observed at posttest (*P* = .14).

At posttest, the BBS scores were significantly higher in the PVT group than in the CPT group (40.36 ± 10.73 vs 34.62 ± 13.56, *P* = .02). The FAC scores were also significantly higher in the PVT group than in the CPT group (3.28 ± 0.76 vs 2.88 ± 0.83, *P* = .01).

### 3.2. Survival analysis (Kaplan–Meier)

**Figure 1.**
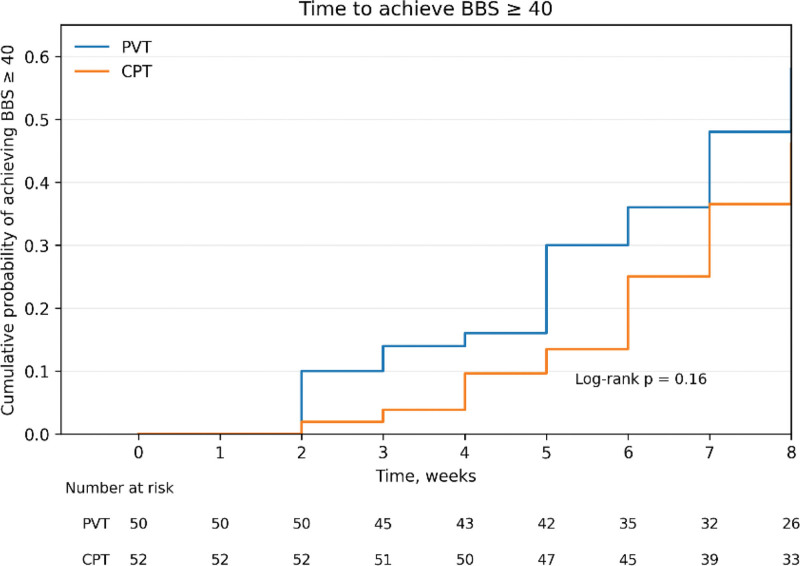
Kaplan–Meier curves for time to achieve BBS ≥ 40 in the PVT and CPT groups, with numbers at risk shown below. BBS = Berg Balance Scale, CPT = conventional physical therapy, PVT = proprioceptive vibration training.

**Figure 2.**
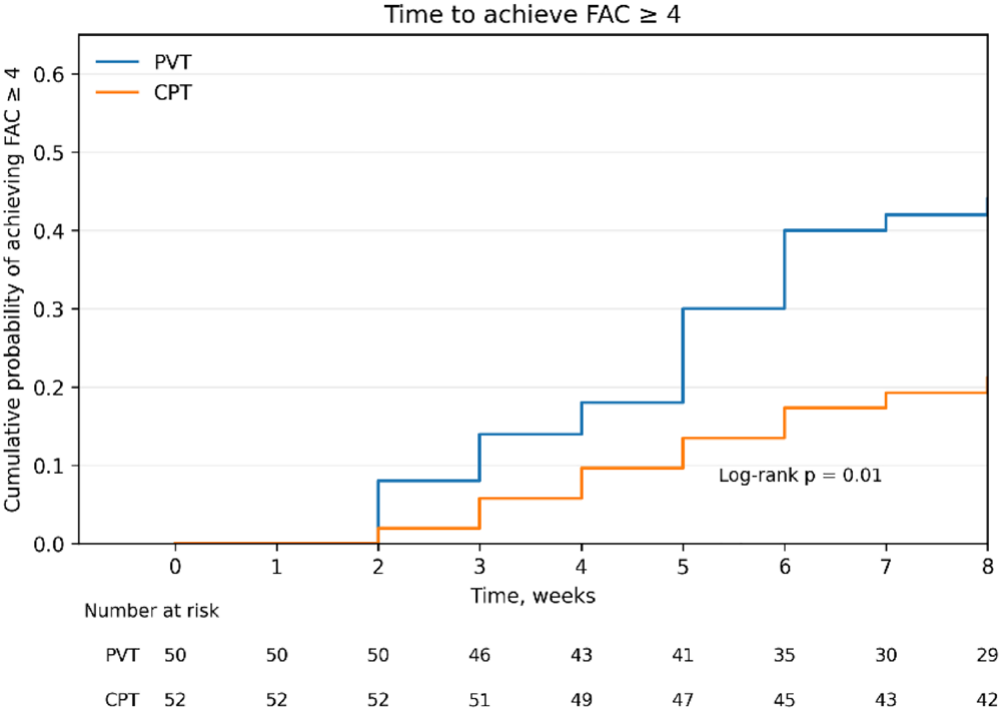
Kaplan–Meier curves for time to achieve FAC ≥ 4 in the PVT and CPT groups, with numbers at risk shown below. CPT = conventional physical therapy, FAC = Functional Ambulation Category, PVT = proprioceptive vibration training.

The mean time to achieve BBS ≥ 40 was 6.46 weeks (95% CI: 5.88–7.03) in the PVT group and 7.09 weeks (95% CI: 6.68–7.50) in the CPT group, with no significant difference between groups (*P* = .16; Fig. 1, Table 3). In contrast, the mean time to achieve FAC ≥ 4 was 6.48 weeks (95% CI: 5.89–7.06) in the PVT group and 7.32 weeks (95% CI: 6.89–7.76) in the CPT group. The log-rank test showed that the PVT group achieved independent walking earlier than the CPT group (*P* = .01; Fig. 2, Table 3).

**Table 3:** Kaplan–Meier analysis and log-rank test results for achieving BBS ≥ 40 and FAC ≥ 4.

Test	Group	Events (n)	Censored (n)	Mean survival time (wk, 95% CI)	χ^*2*^	*P*
BBS	PVT (n = 50)	29	21	6.46 (5.88–7.03)	1.96	.16
	CPT (n = 52)	24	28	7.09 (6.68–7.50)		
FAC	PVT (n = 50)	22	28	6.48 (5.89–7.06)	6.12	.01*
	CPT (n = 52)	11	41	7.32 (6.89–7.76)		

BBS = Berg Balance Scale, CPT = conventional physical therapy, FAC = Functional Ambulation Category, PVT = proprioceptive vibration training.

* *P* < .05.

### 3.3. Cox proportional hazards model results

In the adjusted Cox proportional hazards model for BBS ≥ 40, group allocation was not significantly associated with achievement of the endpoint after adjustment for age, MMSE, sensory status, and baseline BBS (HR = 0.70, 95% CI: 0.40–1.21, *P* = .20). Age, MMSE, sensory status, and baseline BBS were also not significant predictors of achieving BBS ≥ 40.

In the adjusted Cox proportional hazards model for FAC ≥ 4, the PVT group remained significantly associated with earlier achievement of independent walking after adjustment for age, MMSE, sensory status, and baseline FAC (HR = 2.27, 95% CI: 1.09–4.70, *P* = .02). Sensory status was not a significant independent predictor of achieving FAC ≥ 4 (HR = 1.81, 95% CI: 0.63–5.19, *P* = .26). Age, MMSE, and baseline FAC were also not significantly associated with achievement of independent walking (Table 4).

**Table 4:** Adjusted Cox proportional hazards regression results for achieving BBS ≥ 40 and FAC ≥ 4.

Test	Variable	Adjusted HR (95% CI)	*P*
BBS	Group	0.70 (0.40–1.21)	.20
	Sensory	1.09 (0.54–2.18)	.80
	Age	0.99 (0.97–1.02)	.88
	MMSE	1.15 (0.97–1.35)	.08
	Baseline BBS	0.98 (0.96–1.01)	.23
FAC	Group	2.27 (1.09–4.70)	.02*
	Sensory	1.81 (0.63–5.19)	.26
	Age	0.98 (0.95–1.01)	.40
	MMSE	1.06 (0.85–1.32)	.58
	Baseline FAC	0.87 (0.61–1.24)	.44

BBS = Berg Balance Scale, CI = confidence interval, FAC = Functional Ambulation Category, HR = hazard ratio, MMSE = Mini-Mental State Examination.

* *P* < .05.

## 4. Discussion

This study aimed to investigate the effects of PVT on functional ability and the achievement of independent walking in stroke patients, as well as to identify factors influencing walking recovery. The results demonstrated that the PVT group showed significantly greater improvements in both balance, assessed by the Berg Balance Scale (BBS), and gait ability, assessed by the Functional Ambulation Category (FAC), compared to the CPT group. Furthermore, Kaplan–Meier survival analysis and adjusted Cox proportional hazards regression showed that the PVT group was associated with earlier achievement of independent walking compared with the CPT group. This endpoint is clinically meaningful because FAC ≥ 4 corresponds to independent ambulation levels in the original FAC classification and reflects functional walking independence without the need for physical assistance.^[13]^ Although participants with partial sensory impairment showed a higher hazard of achieving independent walking than those with complete sensory loss, this association was not statistically significant in the adjusted Cox model. Therefore, sensory status should be interpreted as a clinically relevant factor rather than an independent significant predictor in the present analysis.

Compared with conventional training, PVT led to greater gains in both balance and gait performance, particularly in dynamic postural control and walking independence. These differences suggest that the proprioceptive stimulation provided by PVT may have accelerated neuromuscular adaptation and functional recovery.

These findings support the effectiveness of PVT as an intervention for improving standing balance and gait ability in stroke patients. Previous studies have suggested that vibration stimulation regulates the activity of spinal anterior horn neurons and enhances cortical excitability, thereby promoting neural reorganization in damaged brain regions.^[5]^ Furthermore, presynaptic inhibition of Ia afferent fibers reduces abnormal spinal reflex excitability, contributing to reduced muscle spasticity and improved functional performance.^[22]^ The present findings are consistent with these mechanisms, suggesting that PVT enhanced afferent sensory input and suppressed abnormal reflex activity, leading to improvements in dynamic balance and gait ability in stroke patients.

Proprioceptive sensation, originating from muscles, tendons, and joints, plays a critical role in perceiving and controlling body position and movement.^[23]^ PVT delivers mechanical vibrations that strongly stimulate proprioceptive receptors such as muscle spindles, Golgi tendon organs, and joint receptors.^[24]^ Similarly, whole-body vibration (WBV) interventions have been reported to promote co-contraction and neuromuscular coordination of the lower extremities, enhancing postural control and responsiveness to external perturbations, ultimately contributing to improvements in balance and gait function.^[19,25]^ The results of this study suggest that the application of PVT shares similar mechanisms and clinical benefits.

In addition, the quantitative analysis using Kaplan–Meier survival curves and adjusted Cox proportional hazards regression further supports the clinical relevance of PVT for walking independence. The PVT group achieved an FAC score of 4, corresponding to independent walking, in an average of 6.48 weeks, approximately 11.5% earlier than the CPT group (7.32 weeks). In the adjusted Cox model, the PVT group had a significantly higher hazard of achieving independent walking than the CPT group, even after adjustment for age, MMSE, sensory status, and baseline FAC (HR = 2.27, 95% CI: 1.09–4.70, *P* = .02). These findings suggest that PVT was associated with earlier attainment of independent walking during the 8-week intervention period.

In contrast, sensory status was not a significant independent predictor of achieving independent walking in the adjusted Cox model (HR = 1.81, 95% CI: 0.63–5.19, *P* = .26). Therefore, the present findings do not support a definitive association between SSEP-based sensory status and earlier walking independence. Although previous studies have suggested that somatosensory function may be relevant to balance and gait recovery after stroke, further studies using more detailed sensory assessments are needed to clarify this relationship.

Consistent with prior studies, the current findings also suggest that vibration interventions may improve balance and walking ability by enhancing proprioceptive input and weight-bearing on the affected side.^[26]^ Furthermore, previous studies have suggested that preserved sensory input may facilitate neuroplastic responses to proprioceptive stimulation and support functional recovery.^[27]^

Previous research has also suggested that sensory function may be related to gait recovery prognosis after stroke. Yoon et al^[28]^ demonstrated that baseline sensory function plays a role in walking performance and recovery among stroke patients, and that somatosensory evoked potential (SSEP) parameters may be associated with gait performance. In the present study, participants with partial sensory impairment showed a tendency toward earlier achievement of independent walking than those with complete sensory loss; however, this association was not statistically significant in the adjusted Cox model. Therefore, the present findings should not be interpreted as confirming sensory status as an independent predictor of walking recovery, but they support the need for further investigation using more detailed sensory assessments.

Most previous studies have primarily focused on short-term outcomes such as improvements in balance or muscle strength, whereas this study is distinct in that it quantitatively analyzed both the achievement of independent walking and the time required to attain it through the application of PVT. In particular, by utilizing Kaplan–Meier survival analysis and adjusted Cox proportional hazards regression, this study objectively evaluated not only whether patients achieved independent walking but also how quickly they reached this clinically meaningful milestone. These findings suggest that PVT may contribute not only to functional improvement but also to the practical enhancement of independence and return to daily life in patients with stroke.

Importantly, early attainment of independent walking is clinically meaningful, as it may shorten the rehabilitation period, promote return to daily activities, and reduce the time and financial burden associated with rehabilitation.^[29,30]^ These findings imply that PVT may contribute not only to individual recovery but also to improving rehabilitation efficiency.

This study has several limitations. First, because the PVT group performed the same conventional exercise program on a vibration platform, the present comparison should be interpreted as conventional training versus conventional training combined with vibration-based proprioceptive stimulation. Therefore, the observed effects may reflect the additive sensory and neuromuscular effects of vibration rather than the independent effect of vibration alone. Second, while this study evaluated functional outcomes such as balance and gait ability, neurophysiological parameters related to neural plasticity and the underlying mechanisms of PVT were not assessed. Future studies incorporating neuroimaging or electromyography are recommended to clarify the physiological mechanisms of PVT. Third, SSEP-based sensory status was dichotomized into partial sensory impairment and complete sensory loss based on the detectability and latency of the P37 response. Although this approach provided a clinically practical classification of somatosensory pathway integrity, it may have oversimplified the spectrum of sensory impairment and did not account for more detailed electrophysiological parameters, such as differences in P37 latency, amplitude, or between-limb SSEP parameters. Future studies should incorporate more detailed SSEP parameters to better characterize sensory impairment and its relationship with walking recovery. Finally, although this study was retrospective in terms of data collection, all evaluations and interventions were implemented within a standardized 8-week inpatient rehabilitation pathway, ensuring consistent timing and procedures across all patients. Nevertheless, residual confounding may still exist due to the retrospective, non-randomized design, and future prospective randomized studies are warranted to establish causal relationships. Despite these limitations, a strength of this study is its use of time-to-event analysis to evaluate not only whether patients achieved independent walking, but also how quickly they reached this clinically meaningful milestone. This approach provides additional insight into the temporal pattern of walking recovery and may help inform rehabilitation planning for patients with stroke.

## 5. Conclusion

This study demonstrated that PVT was associated with improvements in balance and gait ability in stroke patients. Notably, PVT was significantly associated with earlier achievement of independent walking compared with conventional physical therapy after adjustment for age, MMSE, sensory status, and baseline FAC. Sensory status was not a significant independent predictor in the adjusted Cox model. These findings suggest that PVT, as a proprioceptive stimulation-based intervention combined with conventional training, may be a clinically useful strategy to enhance functional recovery and promote independent walking in stroke patients.

## Author contributions

**Conceptualization:** Hyunsik Yoon.

**Data curation:** Hyunsik Yoon.

**Formal analysis:** Hyunsik Yoon.

**Funding acquisition:** Hyunsik Yoon.

**Investigation:** Hyunsik Yoon.

**Methodology:** Hyunsik Yoon.

**Project administration:** Hyunsik Yoon.

**Resources:** Hyunsik Yoon.

**Supervision:** Hyunsik Yoon.

**Validation:** Hyunsik Yoon.

**Writing – original draft:** Hyunsik Yoon.

**Writing – review & editing:** Hyunsik Yoon.
